# Berberine as a potential agent for breast cancer therapy

**DOI:** 10.3389/fonc.2022.993775

**Published:** 2022-09-02

**Authors:** Xiao-Dan Zhong, Li-Juan Chen, Xin-Yang Xu, Yan-Jun Liu, Fan Tao, Ming-Hui Zhu, Chang-Yun Li, Dan Zhao, Guan-Jun Yang, Jiong Chen

**Affiliations:** ^1^ State Key Laboratory for Managing Biotic and Chemical Threats to the Quality and Safety of Agro-products, Ningbo University, Ningbo, China; ^2^ Laboratory of Biochemistry and Molecular Biology, School of Marine Sciences, Ningbo University, Ningbo, China; ^3^ Key Laboratory of Applied Marine Biotechnology of Ministry of Education, Ningbo University, Ningbo, China

**Keywords:** Breast cancer, berberine, apoptosis, autophagic, cell cycle arrest

## Abstract

Breast cancer (BC) is a common malignancy that mainly occurred in women and it has become the most diagnosed cancer annually since 2020. Berberine (BBR), an alkaloid extracted from the *Berberidacea* family, has been found with broad pharmacological bioactivities including anti-inflammatory, anti-diabetic, anti-hypertensive, anti-obesity, antidepressant, and anticancer effects. Mounting evidence shows that BBR is a safe and effective agent with good anticancer activity against BC. However, its detailed underlying mechanism in BC treatment remains unclear. Here, we will provide the evidence for BBR in BC therapy and summarize its potential mechanisms. This review briefly introduces the source, metabolism, and biological function of BBR and emphasizes the therapeutic effects of BBR against BC *via* directly interacting with effector proteins, transcriptional regulatory elements, miRNA, and several BBR-mediated signaling pathways. Moreover, the novel BBR-based therapeutic strategies against BC improve biocompatibility and water solubility, and the efficacies of BBR are also briefly discussed. Finally, the status of BBR in BC treatment and future research directions is also prospected.

## Introduction

Breast cancer (BC) is one of the most common cancers in women and is characterized by a malignant proliferation of mammary tissue (1–3). In 2020, BC has overtaken lung cancer and become the leading diagnosed and death-causing cancer among women all over the world (4–7). BC is a heterogeneous disease and it can be classified into different subtypes based on diverse molecular biomarkers (8). According to the statuses of four molecular biomarkers, namely, estrogen receptor (ER), progesterone receptor (PR), human epidermal growth factor receptor 2 (HER2), and Ki67, BC can be classified into four main intrinsic subtypes: luminal A, luminal B, HER2-enriched, and triple-negative (1, 8). Luminal A subtype is characterized with ER^+^/PR^+^/HER2^-^/Ki-67^low^ biomarkers and low recurrence risk and favorable prognosis. Luminal B subtype is characterized with ER^+^/PR^-^/HER2^+^/Ki-67^high^ biomarkers. HER2-enriched is the third subtype of BC characterized with ER^-^/PR^-^/HER2^+^ biomarkers. Triple-negative BC (TNBC) is the subtype of BC with ER^-^/PR^-^/HER2^-^ biomarkers and the poorest prognosis in clinical studies (7–11).

Currently, there are many methods comprehensively and extensively used in BC therapy. Surgery, radiotherapy, and chemotherapy are the most common therapeutic strategies used to treat all the subtypes of BC in clinic, but most patients would develop drug resistance or relapse later. For the moment, endocrine or single-targeted therapy only has been approved to treat non-TNBC in clinic, and most of them showed poor prognosis and recurrence due to tumor metastasis and drug resistance (11–13), suggesting that a portion of patients develop medicine resistance and experience severe side effects after Western medicine treatment (14). For TNBC, although many novel target therapeutic strategies have been identified and their modulators exhibited good anti-BC activities *in vitro* and *in vivo*, none of them have been approved in clinic due to poor efficacy in clinical trials and potent side effects (11). Therefore, it is extremely eager to seek a more effective conservative and multi-target therapeutic agent that could treat cancer without residual symptoms to patients. Traditional Chinese medicine (TCM) as a therapeutic strategy for varieties of diseases has been used for thousands of years and shows remarkable validation in many diseases. With the development of standardization of TCM, some extracted monomers also show excellent efficacy against different diseases. Among them, alkaloid berberine (BBR) exhibited good efficacy against BC. Here, we summarize the source and biological function of BBR, action mechanisms, and novel therapeutic strategies using BBR against BC. Moreover, the strategies to improve the efficacies of BBR against human BC are also briefly discussed.

## The overview of BBR

**Figure 1:**
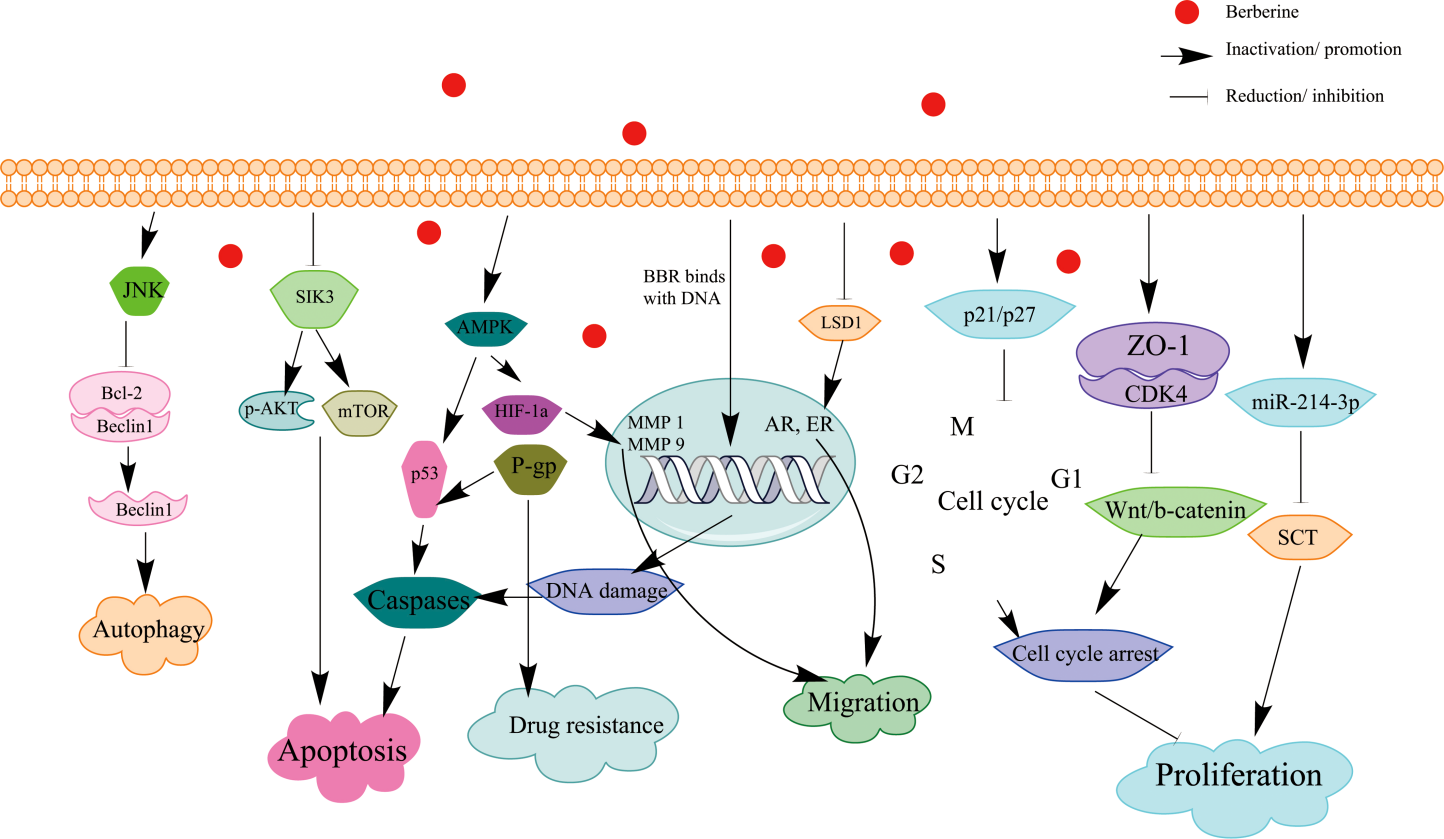
Schematic illustration of the effect of BBR on signaling pathways of apoptosis and cell cycle arrest in BC.

BBR, a pentacyclic isoquinoline compound with a relative molecular weight of 336.37 (**Figure 1**), is a bioalkaloid initially found in the rhizome, bark, and other structures of Chinese herb *Coptis chinensis* Franch, and some berberis plants mainly including *Berberis aristate* DC., *Berberis darwinii* Hook, and *Berberis vulgaris* L (15–17). The later study demonstrated that BBR could be also extracted from some other plants (18). With the aid of UDG glucuronosyltransferase and cytochrome (19), BBR could be transformed into four major types of metabolites: berberrubine, demethyleneberberine, thalifendine, and jatrorrhizine in the liver and intestine *in vivo* (20, 21). Interestingly, the type and quantity of metabolites the metabolites are different in distinct species (22).

BBR, a traditional Chinese medicine, has been used to clear heat and detoxify toxins, promote blood circulation and remove blood stasis, and dispel dampness and cold for over one thousand years (23). Recently, a substantial body of studies showed that BBR has diverse pharmacological effects including anti-hypertensive, anti-oxidative, anti-inflammatory, anti-diabetic, immunosuppressive (24), anti-cardiovascular (21), and neuroprotective activities (3, 6, 25, 26). Therefore, BBR was widely used to treat lots of infectious, metabolic, cardiovascular, and neurological diseases. Notably, BBR also could prevent and treat some different cancers triggered by gene mutations and agricultural hazard factors (27). The mounting evidence suggested that BBR inhibited proliferation as well as induced apoptosis and cell cycle arrest in different cancer cell lines without significant cytotoxicity effect on most non-malignant epithelial cell lines (28–31). Due to its good activity to prevent, inhibit, and reverse the progression of varieties of cancers, BBR has received remarkable interest as a potential anticancer agent in the treatment of many cancers, especially for BC (32).

## The molecular targets of BBR against BC

BBR is a multi-target drug that has been proved effective in treating many cancers and inflammatory diseases. It has been found to combat different cancers *via* binding to diverse molecular targets due to the heterogeneity among different cancers or different subtypes of same cancer. In BC, BBR has also been found to directly bind several target proteins and DNA sequences to exhibit its anticancer activity.

### SIK3

Salt-inducible kinases 3 (SIK3) is an oncogene that plays an important role in BC cells (33). The overexpression of SIK3 promotes BC cell proliferation and growth *via* regulating the cell cycle (34). Meanwhile, BBR is found to function as a SIK3 inhibitor through binding its ATP-binding pocket *via* hydrogen bonding. Further study found that BBR exhibited its anticancer activity partially *via* binding to SIK3 and inducing cell cycle arrest at G1/S cell cycle arrest and apoptosis in BC cells (35).

### Ephrin-B2

Ephrin-B2, a single transmembrane cell surface protein (36), is one of the membrane-bound ligands of the eph receptor family (37). The binding of eph receptor and ephrin-B2 in the membrane activates ephrin-B2 signaling (38). This signaling promotes the cancer cell survival and migration *via* conveying both receptor-expressing cells and ligand-expressing cells (39–41). Ma et al. identified that BBR could directly bind ephrin-B2 and significantly reduce ephrin-B2 level and its downstream proteins (42). The result showed that BBR inhibits the BC survival and migration *via* targeting ephrin-B2 (42).

### LSD1

Histone lysine demethylase 1 (LSD1) is a histone demethylase selectively eliminating the methyl group from histone H3 at H3K4me1/2 and H3K9me1/2 and modulating the transcriptional repression and activation of downstream genes (43, 44). Meanwhile, BC metastasis is associated with the phosphorylation of LSD1 (45). Several studies demonstrated that the stability and degradation of LSD1 could be regulated by ubiquitination and phosphorylation modifications (46). To be specific, reducing ubiquitination levels by deubiquitinase or raising phosphorylation of LSD1 by kinases could inhibit its degradation and increase its stability *via* reducing its ubiquitination level (47). Given that LSD1 could also function as a coactivator *via* demethylating H3K9me1/2 and activating transcription by interacting with androgen receptor (AR) and ER, two receptors that mediated the BC progression (48, 49), it is identified as a promising target for BC therapy (49). As a mainly bioactive product of *C. chinensis* Franch, BBR has exhibited its anticancer activity *via* directly binding to LSD1, inducing the accumulation H3K9me1/2 and suppressing downstream suppressor genes in BC (50–52). Hence, BBR might exhibit its anticancer effect *via* partially targeting LSD1 in BC.

### DNA, TATA box, and poly(A) tails

The nitrogen atom at the 7-position of BBR has a positive charge and this exceptional structure could strongly bind with DNA sequences with negative charge (**Figure 1**) (53, 54). The binding between BBR and DNA would lead to DNA damage in cancer cells *via* regulating the cellular DNA topoisomerase activity (55). Caspase-3 and caspase-9 are proteolytic enzymes that are activated by apoptotic factors, including some target cellular enzymes such as Fas ligand (FasL). BBR could induce the expressions of these apoptotic factors to activate caspase-9 and caspase-3 (56). DNA damage response (DDR) induces cell cycle arrest and apoptosis through the caspase-9 and caspase-3 or Fas/FasL signaling pathway when DNA damage cannot be successfully repaired in time (55, 57) and promotes autophagy to demonstrate its qualities to suppress tumor (58, 59). Therefore, BBR-mediated DDR of cancer cells is also a prospective protein treatment.

Additionally, TATA box is an element that takes part in the process of gene transcription (60). Some results indicated that DNA TATA box and mRNA poly (A) tails played a vital part in the modulation of gene expression, and they are the first and second mainly important targets of BBR to regulate gene transcript (61). In the mechanism, the binding between BBR and TATA box/mRNA poly(A) tails would alter the spatial conformation of these DNA/RNA sequences (60, 61), regulate the transcription of downstream genes, and thus inhibit the BC progression (62). Thus, TATA boxes and poly(A) tails also partially contribute to the anti-BC activity of BBR.

### MicroRNA (miR)-214-3p and SCT

MiR-214-3p is a tumor suppressor functioning by inducing cell apoptosis and G2/M arrest of BC cells (63). *Secretin* (SCT) is an oncogene with an anti-proliferative effect in normal cells but a proliferation-stimulating activity in cancer cells (64). BBR was also found to exhibit its antitumor effects in MCF-7 and MDA-MB-231 BC cells *via* upregulating tumor suppressor microRNA (miR)-214-3p and reducing SCT level (63, 64). Further study showed that BBR exhibited its anti-BC activities *via* directly binding to targeted binding sites within both miR-214-3p and SCT (65). In addition, BBR could raise the miR-214-3p expression and reduce SCT level *via* β-catenin–mediated inactivation of telomerase activity (66).

## The BBR-mediated pathways of against BC

### BBR suppresses proliferation and migration of BC cells *via* ZO-1 mediated Wnt/β-catenin signaling pathway

Zonula occludence-1 (ZO-1) is a key molecule tightly attached to other proteins, such as Wnt/β-catenin. Wnt/β-catenin is a membrane-linked protein that could be activated *via* dissociating from the membrane. The activation of Wnt/β-catenin signaling promotes tumor cell proliferation and migration (67). ZO-1 was found to be activated by BBR, which lead to enhancing the binding of ZO-1 and the cyclin-dependent kinase 4 (CDK4) and reducing CDK4 entry into the nucleus (68, 69). Then, the free Wnt/β-catenin level was also downregulated, which suppresses cancer cell proliferation *via* induced cell cycle arrest (70, 71). The present study also demonstrated that BBR inhibited the proliferation and migration of BC cells partially *via* inactivating the ZO-1–mediated Wnt/β-catenin signaling pathway.

### BBR induced cell cycle arrest of BC cells through upregulating p21 and p27

P21 and p27 are the key CDK inhibitors (CDKIs) that induce apoptosis and cell cycle arrest in varieties of cells (72). The increase of mRNA and protein expression levels of p21 and p27 is beneficial to inhibit the expressions of diverse cyclins including cyclin D1, cyclin E, cyclin K2, cyclin K4, and cyclin K6 (73). The CDKIs would modulate cell cycle arrest in the G1 phase and induce apoptosis in different cancer cells.

Several studies proved that BBR could modulate different tumor suppressor genes (including p21 and p27) to induce cytotoxicity in BC cells, and reducing the two protein levels strongly clocks up the growth of different tumor types including BC (73). Further study indicated that BBR could induce G1 phase arrest and thus inhibit cell proliferation *via* upregulating p21 and p27 (74).

### BBR sensitizes chemical agents and overcomes drug resistance of BC cells *via* activating AMPK signaling

Adenosine monophosphate–activated kinase (AMPK), a sensor of energy status, plays an extremely primary part in cellular energy homeostasis and regulates the drug resistance of BC (75, 76). It is activated by hypoxia to compensate for the oxygen reduced by mitochondria respiration (77). Hypoxia-inducible factor-1alpha (HIF-1α), the primary regulator of cell response to hypoxia (78, 79), has the function to increase proliferation and drug resistance of some cancers including BC *via* regulating metabolic enzymes (80, 81). P-glycoprotein (P-gp) is a critical obstacle to reduce drug accumulation in cancer eradication (82). As a tumor suppressor protein (83), p53 can be activated by being modified by the phosphorylation of multiple protein kinases at multiple sites (84). Interestingly, HIF-1α, P-gp, and p53 are downstream genes of AMPK signaling, and BBR exhibits its anti-BC activity *via* AMPK-mediated reducing level of HIF-1α and P-gp and phosphorylating p53 (85, 86).

Doxorubicin (DOX) is the most frequently used chemical agent to treat BC in clinic. However, its effectiveness is often reduced in a hypoxic environment (87). Pan et al. found that the activation of AMPK signaling was responsible for lowering the sensitivity of BC cells to DOX. To be specific, AMPK was activated by a hypoxic environment, which upregulated the phosphorylated AMPK (p-AMPK) and HIF-1α. Then, HIF-1α raised P-gp level and thus improved the DOX sensitivity of BC cells. BBR also could overcome DOX resistance *via* AMPK in a time- and dose-dependent manner. Further study showed that low-dose BBR enhanced cytotoxicity and sensitized DOX sensitivity *in vivo via* inhibiting the AMPK pathway, whereas high-dose BBR would restrain the activation of AMPK and affect HIF-1α downregulation, which induces p53 activation that led to cell death and apoptosis (88). In conclusion, BBR enhanced sensitivity to chemical agents and drug resistance of BC mainly through activating AMPK signaling cascades (89).

### BBR inhibits TPA-induced PKC-α signaling and thus reduces the levels of *MMP-1* and *MMP-9* in BC

Phorbol ester 12-*O*-tetradecanoylphorbol-13-acetate (TPA) was found to promote tumor invasion and migration in BC (90). Matrix metalloproteinases (MMPs) are key enzymes that could regulate the cellular microenvironment (91). Matrix metalloproteinase-1 (*MMP-1*) is the principal LCC-secreted factor that enhances the tumor-promoting traits (92). Matrix metalloproteinase-9 (*MMP-9*) is an enzyme which belongs to the MMP family, and its activity was related with different stages of carcinoma progression (93, 94). Several studies showed that the *MMP-9* level is positively correlated with a higher tumor grade in BC tissue (95), and TPA mediated tumor invasion and migration by upregulating expression of *MMP-1* and *MMP-9* in BC cells (96, 97). Protein kinases C (PKCs) are a set of serine/threonine kinases involved in the tumor progression in BC (98). Protein kinase C (PKC-α) can be activated by TPA and promote *MMP-1* and *MMP-9* expressions (99). Meanwhile, it may be elevated in patients with lower ER levels. Further studies indicated that the TPA dose-dependently enhanced the expression levels of *MMP-1* and *MMP-9 via* inducing phosphorylation of PKC-α (99), and BBR administration could suppress TPA-induced proliferation and formation of BC cells *via* blocking PKC-α/MMP signaling (100, 101). The result demonstrated that BBR also exhibited its anticancer activity *via* inhibiting the abnormal expressions of *MMP-1* and *MMP-9* during invasion and proliferation of cancer (102, 103). Here, PKC-α/MMPs signing may be also a potential signaling that mediated the anticancer activity of BBR.

## BBR-based combined strategies for BC treatment

### BBR and curcumin

Curcumin (CUR) is a natural phenolic product distilled from the rhizome of *Curcuma Longa* L. and its main functions are similar to BBR (104). Both natural products are famous for their multiple pharmacological properties such as anticancer and anti-inflammation activities (105, 106). Many studies revealed that CUR and BBR exhibited outstanding anticancer properties with low toxicity in multiple cancer types (69, 107). Apoptosis and autophagy have been shown to interrelate by many complex mechanisms (108). Clinical analysis revealed that CUR and BBR have the pharmacological capability to mediate autophagic and apoptosis in multiple cancer cells *via* diverse signaling pathways, such as p-Jun N-terminal kinase (JNK) signaling, Beclin1/Bcl-2 signaling, and ERK signaling.

JNK is found to mediate autophagy in response to cell stresses *in cellulo* (109). Beclin1, a protein only consisting of Bcl-2 homology 3 domain, is also a vital initiator of autophagy (110). Bcl-2 and Bax belong to Bcl-2 family proteins. Bcl-2 protein is anti-apoptotic, whereas Bax protein is pro-apoptotic (111). Extracellular signal–regulated protein kinases (EKRs) play important roles in cell proliferation and apoptosis, and they could block apoptotic and induce proliferation by enhancing the expressions of pro-survival genes and reducing associated genes, such as *Bcl-2* and *Bax* (112). Mounting evidence showed that the JNK activation increased phosphorylated Bcl-2 level and dissociation of the Beclin1/Bcl-2 complex (109). Then, the dissociated Beclin1 would induce autophagy. He et al. confirmed that combined therapy using CUR and BBR increases phosphorylated Bcl-2 *via* activating the JNK pathway (108, 113, 114).

ERK is a signaling regulator modulating the proliferation, growth, and survival in most of cell lines (114). However, its activation is also found to mediate cell death in some cell types (115). CUR or BBR treated alone has been found to activate ERK signaling *via* phosphorylating ERK in many cancer cells, whereas their combined use significantly enhanced ERK phosphorylation and then raised Bax level and reduced the expression of Bcl-2. Meanwhile, the upregulated Bax/Bcl-2 ratio would inhibit the proliferation of cancer cells *via* inducing apoptosis (11).

All the results strongly suggested that the combined treatment using BBR and CUR significantly improved anticancer efficacy *via* inducing autophagy and apoptosis of BC cells through the JNK/Beclin1/Bcl-2 pathway and ERK signaling. This combined strategy may be a promising medicine to treat BC.

### BBR and emodin

Emodin (EMO) is a natural anthraquinone compound extracted from several medicinal plants and it has been found to exhibit anticancer effects in BC by regulating several signaling pathways (116). SIK3 belongs to the AMP-activated protein kinase family, and it is required for the mTOR/AKT signaling pathway to mediate the proliferation of BC cells. Serine/threonine kinase (AKT), a regulator modulating the cell cycle progression and survival, is also a factor involved in anticancer effect in BC cells (117). The activation of AKT signaling contributes to tumor progression and drug resistance in various types of cancer (118). The mammalian target of rapamycin (mTOR), a highly conserved kinase, is an important regulatory factor to control translation and proliferation in different cancer cell lines. SIK3 is overexpressed and enhances the phosphorylation of AKT in the BC. Then, the phosphorylated AKT is activated, promotes G1/S cell cycle progression, and finally leads to cell proliferation and survival of cancer cells (119). BBR and EMO were identified as potent SIK3 inhibitors in BC *via* directly binding to the ATP binding pocket of SIK3 by hydrogens. One of the possible mechanisms was that BBR and EMO inhibited the expression of SIK3 *via* inactivating the AKT signaling pathway and inducing G1/S cell cycle arrest and apoptosis of BC cells (30). In addition, some results revealed that the combined therapy using BBR and EMO significantly decreased the phosphorylation of AKT compared with either a single treatment in BC cells (35). It is worth mentioning that the combined therapy did not affect the growth of non-tumorigenic cells, suggesting that this strategy may be an effective method to treat BC.

Cancer cells consume a lot of glucose to maintain their persistent proliferation, and mTOR-induced aerobic glycolysis provides the source for this process (120). mTOR is also a kinase regulated by SIK3 and plays crucial roles in BC proliferation. *Phosphorylation of p70 S6 kinase 1* (*p-S6K1*) and *phosphorylated E4 binding protein 1* (*p4EBP1*) are two downstream targets of mTOR, and SIK3 regulates the mTOR signalling pathway and promotes the proliferation of BC cells *via* promoting phosphorylation of them at residues T389 and T37/46, respectively (63, 121). Meanwhile, EMO and BBR alone or combined administration could inhibit mTOR signaling and growth cell cycle arrest, and apoptosis of BC cells *via* downregulating phosphorylation levels of p-S6K1 (T389) and p4EBP1(T37/46) (35). Current results suggested that the combination of BBR and EMO inhibits proliferation and growth in BC cells. To sum up, BBR and EMO suppress growth and proliferation through the inactivation of SIK3-induced mTOR and AKT signaling pathways.

## Conclusions

BBR has exhibited distinct pharmacological activities in different diseases. In this mini-review, the source and biological function of BBR have been summarized, and the anticancer effects of BBR on BC and its underline mechanisms have been also systematically described. The review demonstrated that BBR exhibited its anti-BC effects through binding to the effector proteins/miRNA/DNA regulatory sequences and thus inhibiting multiple cancer-related signaling (**Figure 1**). Notably, there are several reported molecular targets for BBR in other cancers such as receptor retinoid X receptor alpha (RXRα) (71), protein tyrosine phosphatase 1B (PTP1B) (122), TNF receptor–associated factor 6 (TRAF6) (123), and angiotensin-converting enzyme (ACE), and these proteins also play important roles in BC; further studies are necessary to investigate the roles of these targets in BBR-mediated anti-BC effects. BBR not only could directly bind to oncogenes ephrin-B2, SIK3, and LSD1 and inactivated their functions in BC but also modulated the transcription of some cancer-related genes *via* directly binding to their cis-acting elements. In addition, BBR has been found to inhibit cancer cell proliferation and invasion *via* several signaling pathways (**Table 1**).

**Table 1:** The reported pathways and molecular targets regulated by BBR in different BC cell lines.

Dosage	Function	Type of cell lines	Study model	References
1, 2, and 4 mg/kg	BBR act directly on the poly(A) tail	The male mice	*In vitro*	(124)
MDA-MB-231: 0, 6.25, 12.5, and 25 µM; MDA-MB-468: 0, 3, 6, and 12 µM; MDA-MB-453: 0, 2.5, 5, and 10 µM	Induce cell cycle arrestReduce the expression of cyclin D and Cyclin E	MDA-MB-231, MDA-MB-468, MDA-MB-453, and BT-549 cells	*In vitro*	(125)
100 mM	Suppress the expressions of *MMP-1* and *MMP-9*	MCF-7 and MDA-MB231	*In vitro*	(99)
BBR: 0–40 µMEMO: 0–40 µM	BBR binds with SIK3 to inhibit SIK3 activityInduce cell cycle arrest at G1/S cell cycle arrest and apoptosis	MCF-7, T47D, MDA-MB-468, and MDA-MB-231 cells	*In vitro*	(35)
5, 10, and 20 μmol/L	Inhibit the AMPK pathway to induces cell death and apoptosis	MCF-7/MDR cells	*In vitro*	(88)
25 and 50 µM	Upregulate miR-214-3pReduce SCT	MCF-7 and MDAMB-231 cells	*In vitro*	(63)
0–16μg/ml	Reduce nuclear CDK4Downregulate Wnt/β	MCF-7 cells	*In vitro*	(68)

Although BBR exhibited potential anti-BC activity in preclinical studies, there are several predicaments to overcome before it was been advanced into clinical treatment against BC. Firstly, several studies showed that BBR exhibited low toxicity to healthy cells and human beings (126, 127). It could also lead to some adverse events, such as constipation and nausea (128, 129). In addition, some patients injected with BBR through intramuscular and intravenous had presented allergic reactions (130). Moreover, the poor oral bioavailability and low water solubility of BBR could reduce its anticancer activities (131). Currently, several strategies have been introduced to improve the efficacy of BBR against BC and reduce side effects *in vivo*. Combined therapy using BBR and other natural compounds is proved to significantly improve the anti-BC activity of BBR *via* inducing more effective apoptosis and reducing the dose of BBR (65, 103). Apart from combined therapy, conjugating BBR with other chemical agents is also an efficient strategy to improve the anticancer activity of BBR for BC therapy (132, 133). Qin et al. found that a conjugator *via* linker BBR to platinum (II) complex significantly improved the *in vitro* and *in vivo* anti-BC activity (134). In addition, drug delivery using nanocarriers is widely used to improve cell penetration, biocompatibility, and *in vivo* efficacy, and this method is also an applicant for improving bioavailability and the efficacy of BBR against BC (135–137). Moreover, chemical modification is also a potent strategy to improve cell penetration, and water soluble anti-tumor activity of BBR and several BBR derivatives have been designed and exhibited better anti-BC efficacy than their lead compound BBR (132, 138–141).

In conclusion, although some studies showed that BBR induced apoptosis of some BC cell lines and sensitized BC cells to chemotherapy *via* interfering with some pathways, the detailed mechanisms of the BBR are unclear, and more potential anti-tumor pathways or targets are yet to clarify due to the heterogeneity of BC. Further research would make a thorough inquiry regarding the clinical effect of BBR and whether the combined therapy/nanocarriers/chemical modifications could show a more valid effect on patients and reduce the side effects. Meanwhile, it is imperative to consider the treatment cycle and degree of BBR in clinic. In short, further studies are warranted to define the therapeutic role of BBR as an anticancer drug in BC.

## Author contributions

X-DZ: Writing-Original draft preparation. L-JC and C-YL: Prepared figures and Tables. X-YX, Y-JL, FT, and M-HZ: Resources, Conceptualization, Investigation and Validation, Writing reviewing and editing. DZ, G-JY and JC: Supervision, Funding acquisition, Conceptualization, Writing reviewing and editing. The authors contributed to the data preparation and drafted and revised the manuscript. All authors contributed to the article and approved the submitted version.

## Funding

This work is supported by the National Natural Science Foundation of China (31972821), the General Scientific Research Project of Education of Zhejiang Province (Y202147351), the Starting Research Fund of Ningbo University (421912073), Student Research and Innovation Program of Ningbo University (2022SRIP1818), and State Key Laboratory for Managing Biotic and Chemical Threats to the Quality and Safety of Agro-products (2010DS700124-ZZ2008).

## Conflict of interest

The authors declare that the research was conducted in the absence of any commercial or financial relationships that could be construed as a potential conflict of interest.

## Publisher’s note

All claims expressed in this article are solely those of the authors and do not necessarily represent those of their affiliated organizations, or those of the publisher, the editors and the reviewers. Any product that may be evaluated in this article, or claim that may be made by its manufacturer, is not guaranteed or endorsed by the publisher.
